# Management of peri‐implant soft tissue dehiscence with combined prosthetic‐surgical approach: A case report

**DOI:** 10.1002/cap.70039

**Published:** 2025-11-19

**Authors:** Omran Bishbish Zeino, Yoon Jeong Kim

**Affiliations:** ^1^ Department of Periodontics Loma Linda University School of Dentistry Loma Linda California USA

**Keywords:** connective tissue, dental implants, patient satisfaction, soft tissue dimension, soft tissue grafting

## Abstract

**Background:**

This case report presents the management of an esthetic complication of a peri‐implant soft tissue dehiscence (PSTD) through a combined prosthetic‐surgical approach.

**Methods and Results:**

A 53‐year‐old healthy Hispanic male presented to our practice for the treatment of an implant esthetic complication. A diagnosis of PSTD class III C was established. The abutment and crown were first modified to accommodate the tissue graft and support the coronally advanced flap (CAF). Then, a CAF with tuberosity connective tissue graft was performed. A definitive abutment and crown were fabricated 8 months after healing. Significant improvement of the PSTD, improvement of the peri‐implant soft tissue dimensions, and patient satisfaction have been achieved.

**Conclusion:**

A combined prosthetic‐surgical approach constitutes a valid treatment modality for PSTD class III C where there is abundant interproximal tissue available.

**Key points:**

- **Integrated treatment approach**: A combined prosthetic‐surgical technique offers an effective solution for managing peri‐implant soft tissue dehiscence (PSTD), ensuring improved tissue thickness and stability.
- **Clinical considerations**: The bucco‐lingual implant position and interproximal tissue quality are key factors in determining the optimal treatment strategy.
- **Predictable outcomes**: Coronally advanced flap combined with connective tissue grafting can enhance esthetic and functional results for management of PSTD.

**Plain language summary:**

Peri‐implant soft tissue complications can affect both the function and appearance of dental implants. This case study explores an approach that combines surgical and prosthetic techniques to improve the gingival tissue surrounding an implant. A 53‐year‐old patient had an esthetic concern due to gum recession around his implant. To correct this, his dental crown and abutment were adjusted. Then, a gingival grafting procedure to reposition the gingival tissue and enhance its thickness was performed. After healing for eight months, the implant was permanently restored with a final crown. The results showed significant improvements in gingival tissue health, thickness, and appearance, leading to patient satisfaction. The findings highlight how combining surgical techniques with prosthetic adjustments can help manage similar cases, offering a predictable solution to improve both the appearance and stability of dental implants.

## INTRODUCTION

Peri‐implant soft tissue dehiscence (PSTD) is defined as an alteration of the peri‐implant soft tissue architecture characterized by an apical discrepancy of the peri‐implant mucosal margin with respect to its ideal position.^1^ This case report presents the management of a PSTD with an esthetic complication through a combined prosthetic‐surgical approach. The objectives of treatment were to improve the level of the peri‐implant mucosal margin, optimize the dimensions of the peri‐implant soft tissue, and ensure patient satisfaction. CARE reporting guidelines were followed during the preparation of this manuscript.^2^

## MATERIALS AND METHODS

### Clinical presentation

**FIGURE 1:**
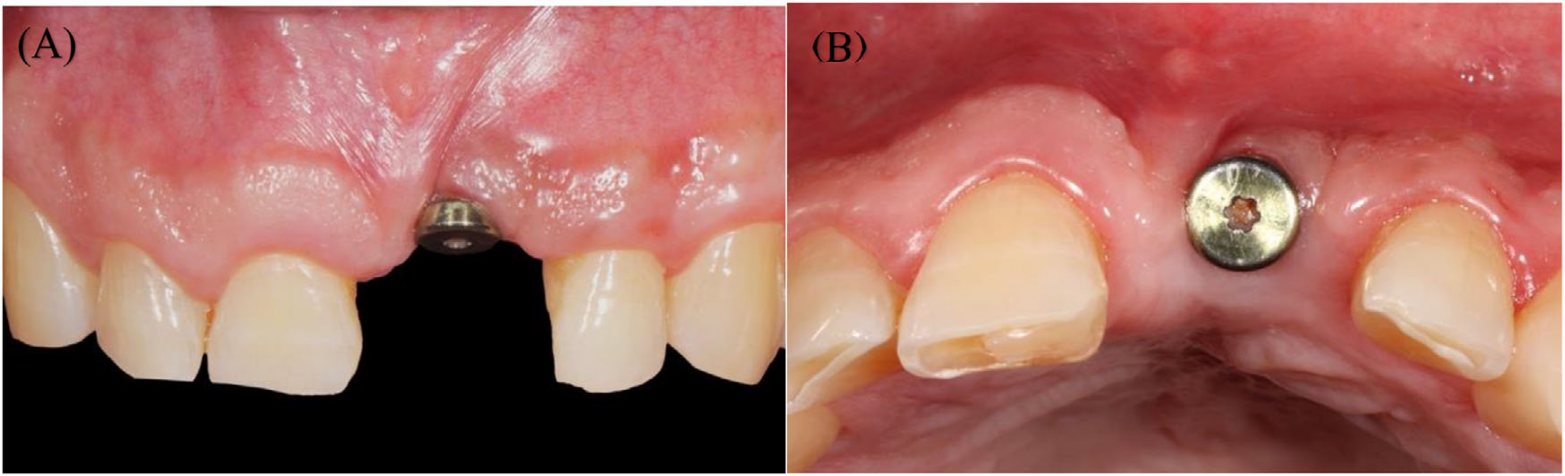
Initial presentation with healing abutment placed on implant #9 by the referring provider. (A) Frontal view showing interproximal tissue loss on teeth #8, 10, and CEJ exposure. (B) Occlusal view.

**FIGURE 2:**
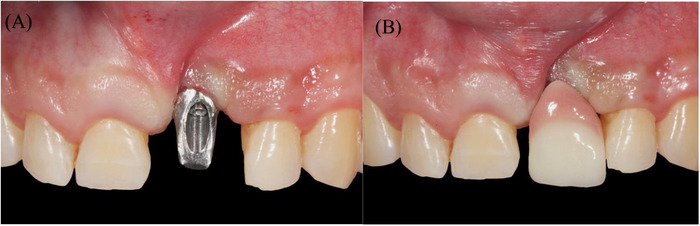
Frontal view of: (A) the final abutment, and (B) the crown, which were made by the referring dentist but never delivered due to esthetic concerns.

A 53‐year‐old healthy Hispanic male was referred to our practice in Redlands, California, for consultation and treatment of an esthetic complication involving an existing dental implant in the left maxillary central incisor area #9. The referring provider placed the implant immediately after extracting tooth #9 and allowed the site to heal for four months. Following second‐stage surgery, the referring provider attempted to restore the case. Although the definitive restoration was fabricated, it was not accepted by the patient due to to esthetic limitations and patient dissatisfaction (Figures 1 and 2).

**FIGURE 3:**
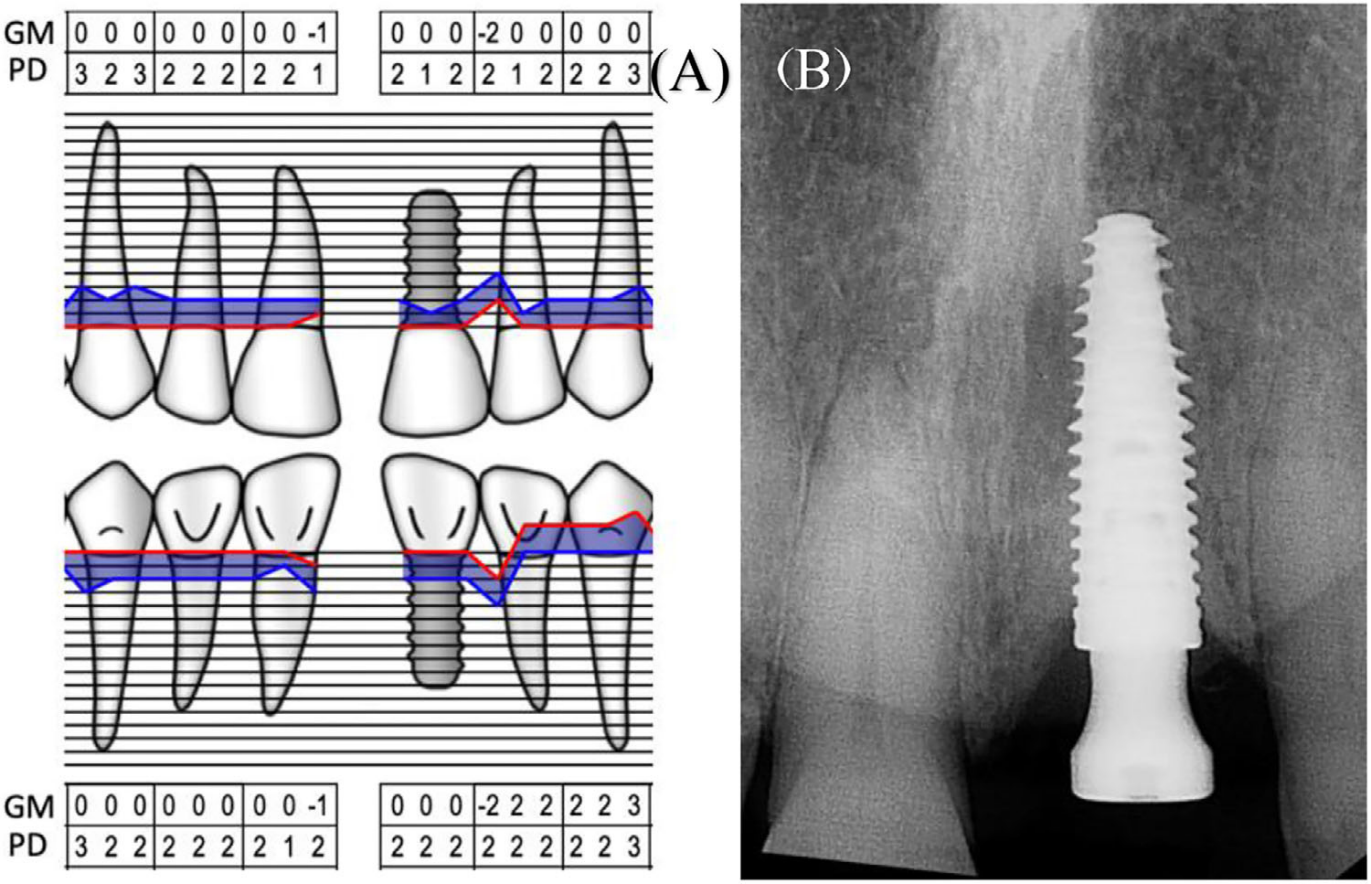
(A) Periodontal chart, maxillary anterior area. (B) Peri‐apical radiograph showing minimal crestal bone level changes.

Clinical evaluation revealed no interproximal bone loss around the implant, no bleeding on probing, and probing depths (PDs) of <3 mm (Figure 3). A high labial frenum attachment was observed, impinging on the implant's facial gingival margin during lip retraction (Figure 2).

The peri‐implant phenotype was classified as thin, with a mucosal margin thickness of <2 mm and a narrow band of keratinized mucosa <2 mm.^3^ A discrepancy in the mucosal margin of the #9 implant, relative to its ideal position, was evident. Consequently, a diagnosis of PSTD was established.^1^

**FIGURE 4:**
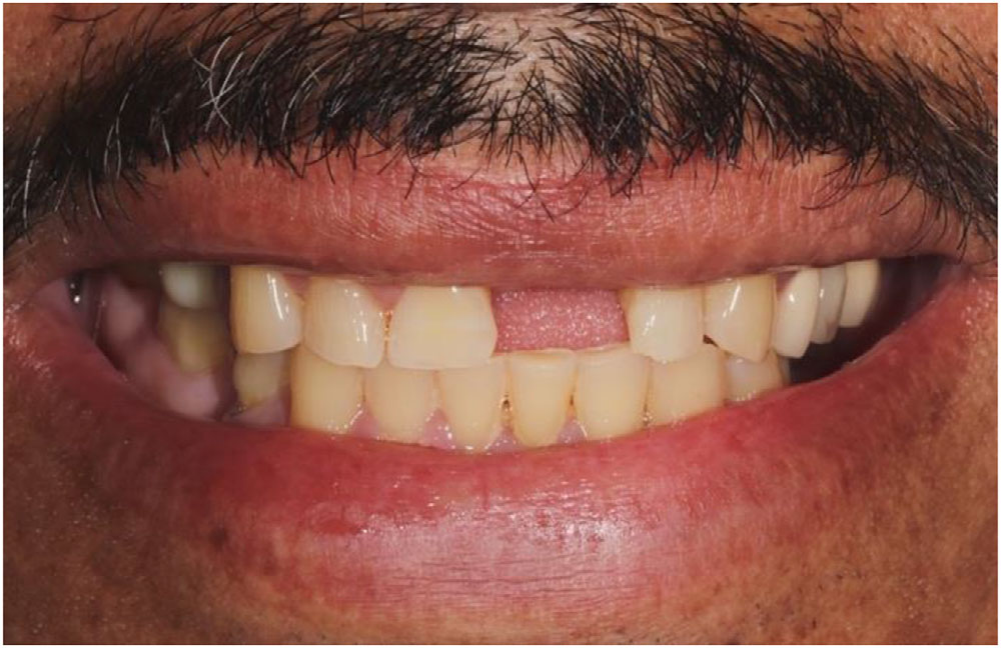
Frontal view shows a low smile line.

Space analysis revealed a discrepancy of 2 mm between the mesial‐distal dimension of #9 space compared to the tooth #8 crown (Figure 2). The patient reported having a midline diastema before the extraction of #9. Smile analysis indicated a low smile line (Figure 4).

Evaluation of adjacent teeth #8 and #10 showed short clinical crowns, a thick periodontal phenotype, and interproximal attachment loss of 2 mm on the mesial of tooth #8 and 4 mm on the mesial of tooth #10. No PD greater than 3 mm or signs of active periodontal disease were detected. Reduced papilla height with exposed CEJ was noted on the mesial surface of both #8 and #10 (Figure 1A).

**FIGURE 5:**
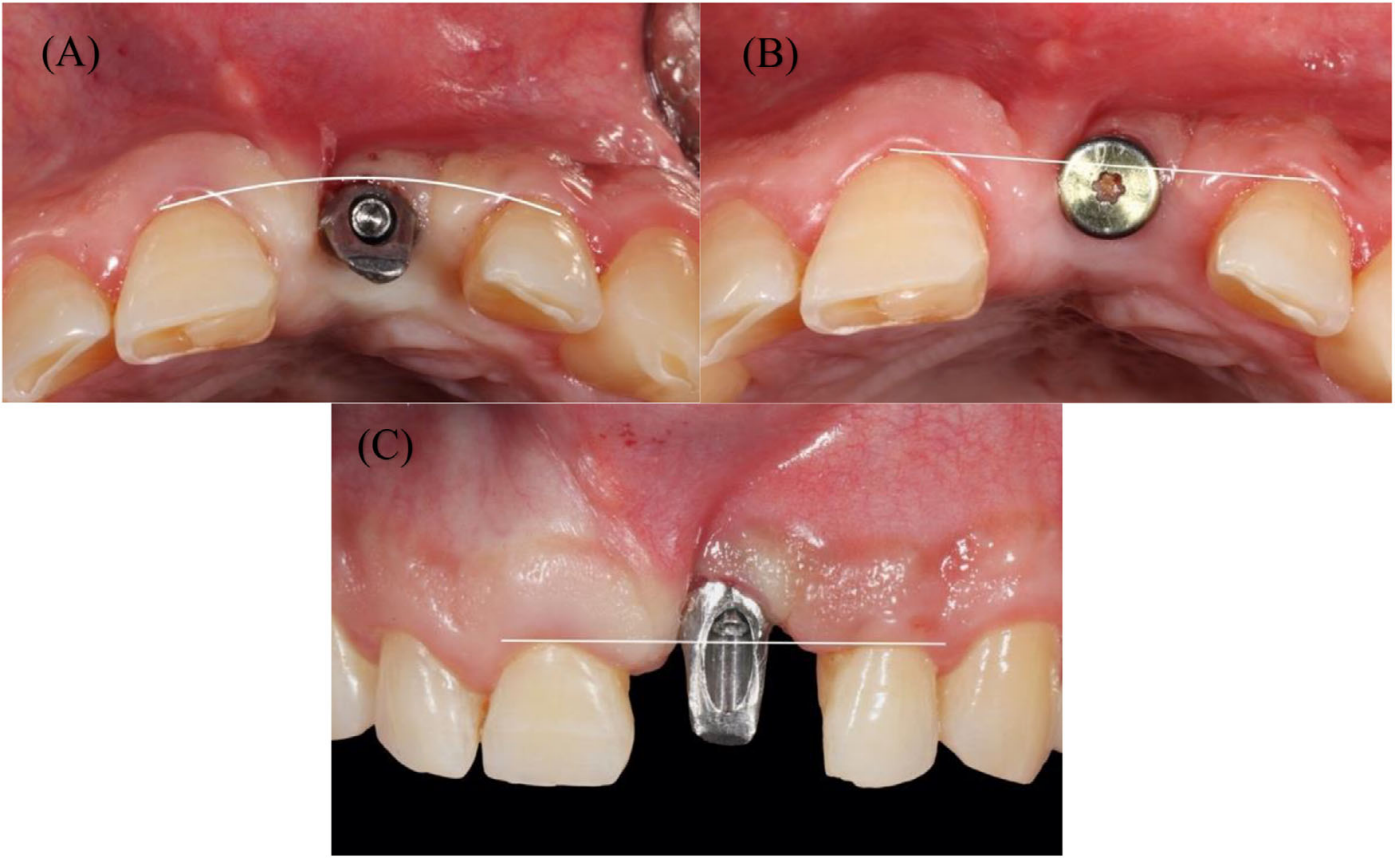
(A) The implant‐supported crown profile was located outside the imaginary curve line that connects the profile of the adjacent teeth at the level of the soft tissue margin. (B) The head of the implant is more palatal than the imaginary straight line connecting the profile of the adjacent teeth. (C) The height of the distal papilla is <1 mm coronal to the ideal position of the soft tissue margin of the implant‐supported crown.

Further assessment using Zucchelli's classification for PSTD was performed.^4^ The implant‐supported crown profile was positioned outside the imaginary curve line connecting the profile of the adjacent teeth at the level of the soft tissue margin. Additionally, the head of the implant was located more palatally than the imaginary straight line connecting the profile of the adjacent teeth. The distal papilla height measured <1 mm coronal to the ideal soft tissue margin of the implant‐supported crown (Figure 5). Based on this evaluation, the defect was classified as Class III, Subclass C.^4^

### Case management

Findings, diagnosis, and treatment options—including implant removal, orthodontic treatment, and esthetic crown lengthening of adjacent teeth—along with treatment limitations were discussed with the patient. The patient was primarily focused on addressing the defect of the existing implant and declined any intervention to alter the shape of adjacent teeth or modify the gingival margin levels at this point. According to Zucchelli et al.,^4^ the recommended treatment for PSTD Class III C involves soft tissue augmentation in a submerged fashion. However, in this case, the implant did not yet have a final restoration, and there was a significant amount of interproximal soft tissue present, characterized by wide and thick papillae. Therefore, a surgical‐prosthetic approach was proposed to manage the case without submerging the implant. This approach allowed for immediate tooth replacement and significantly reduced overall treatment time.

The patient agreed to the treatment plan and understood the treatment limitations. The patient was fully aware of the fact that a symmetric and harmonious peri‐implant mucosal margin is not likely to be achieved without altering the gingival margin of adjacent teeth. A written consent was obtained.

**FIGURE 6:**
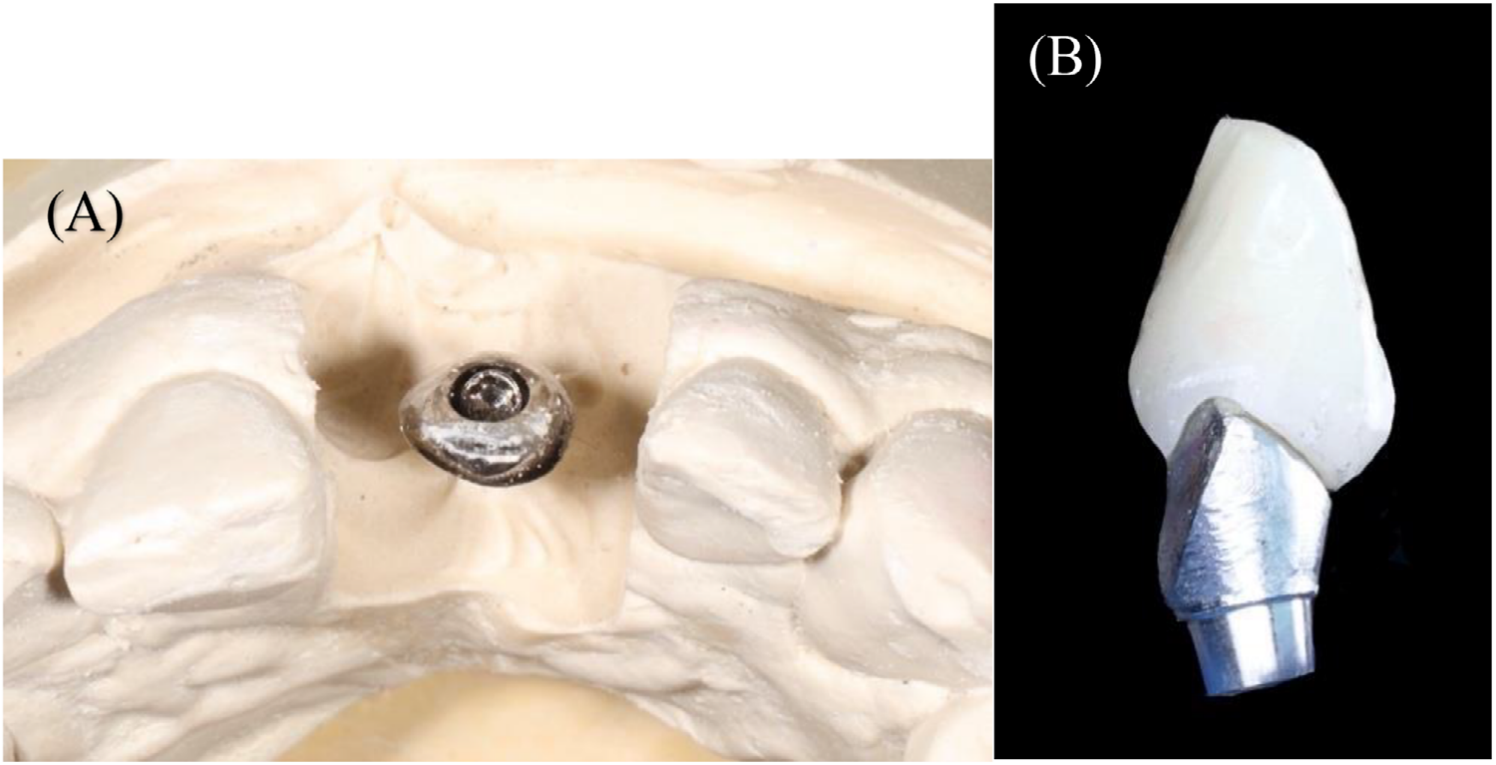
Abutment and crown modification to serve as a provisional restoration during healing.

Modification of the existing abutment and crown using rotary instruments was performed on the cast. The goal was to create a subgingival flat/concave wide surface that provides space for the graft volume, followed by a slightly convex surface at the level of the future peri‐implant mucosal margin to support the future flap margin of the coronally advanced flap (CAF) (Figure 6).

The abutment was connected to the implant, and local anesthesia was performed using 2% lidocaine with 1/100,000 epinephrine. A horizontal incision for a CAF was performed on the facial aspect of the abutment, followed by two slightly divergent vertical incisions that were extended to the alveolar mucosa. A split‐thickness flap was carefully elevated, ensuring that the connective tissue on the implant surface was preserved. The flap was released through a deep incision made parallel to the bone, followed by a superficial incision with the blade aligned parallel to the external mucosal surface. This allowed the flap to passively extend to its final position, which was limited by the height of the interproximal papillae. Additionally, this approach released the frenum pull from the flap margin.

**FIGURE 7:**
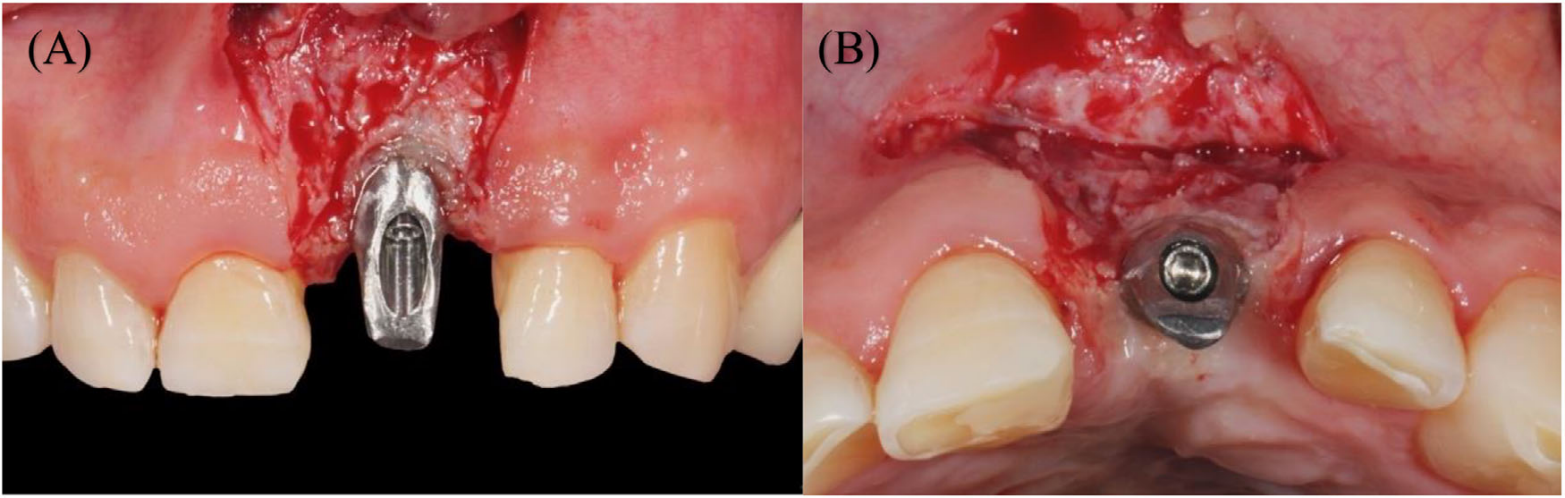
(A) Flap incisions and partial thickness flap. (B) Occlusal view shows interproximal tissue de‐epithelialization.

**FIGURE 8:**
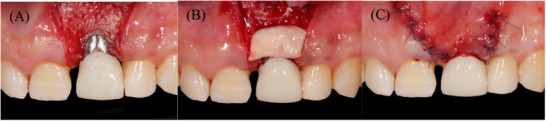
(A) Temporary crown cemented. (B) Connective tissue graft in place. (C) Final flap position and sutures.

The anatomical papillae were de‐epithelialized on both the buccal and occlusal aspects to enhance the vascular bed (Figure 7). The provisional crown was cemented onto the abutment, ensuring no contact with the soft interproximal tissue. A 10 mm long and 6 mm wide area in the right maxillary tuberosity was de‐epithelialized using a diamond bur and a handpiece. Subsequently, a 1.5 mm thick tuberosity connective tissue graft (tCTG) was harvested from the area. A collagen sponge was placed at the donor site and secured using 3/0 chromic gut sutures to achieve hemostasis. The tCTG was stabilized with simple interrupted sutures using 5/0 chromic gut to the de‐epithelialized anatomical papillae on the coronal aspect, and to the periosteum on its apical aspect. Then, the flap was sutured to the final position by performing simple interrupted sutures at the level of the vertical releasing incisions, and between the surgical and the anatomical papillae (Figure 8).

Postoperative care included ibuprofen 800 mg for pain as needed, and chlorhexidine rinses twice daily for one week. The patient was advised to follow a soft food diet and avoid oral hygiene practices on the surgical site for three weeks. Starting in the fourth week, the patient was instructed to resume gentle brushing with a soft toothbrush.

## RESULTS

**FIGURE 9:**
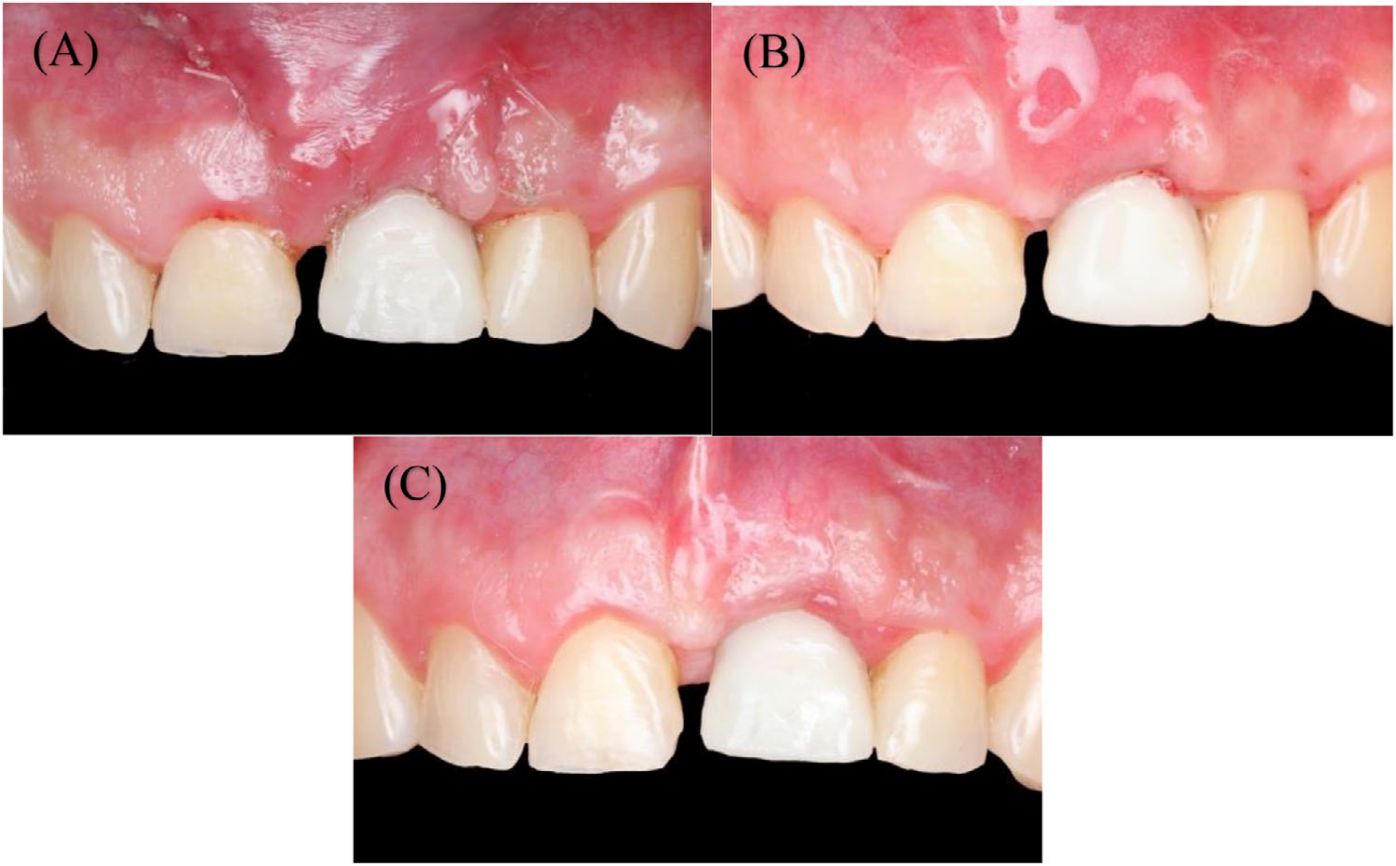
(A) 3 weeks postoperative. (B) 6 weeks postoperative. (C) 16 weeks postoperative.

Postoperative follow‐up appointments were scheduled at 3, 6, and 16 weeks (Figure 9), during which oral hygiene instructions were reinforced and supragingival debridement was performed as needed. At that time, the patient was satisfied with the results and expressed interest in exploring options to close the diastema. He was referred to his referring provider to discuss restorative solutions.

**FIGURE 10:**
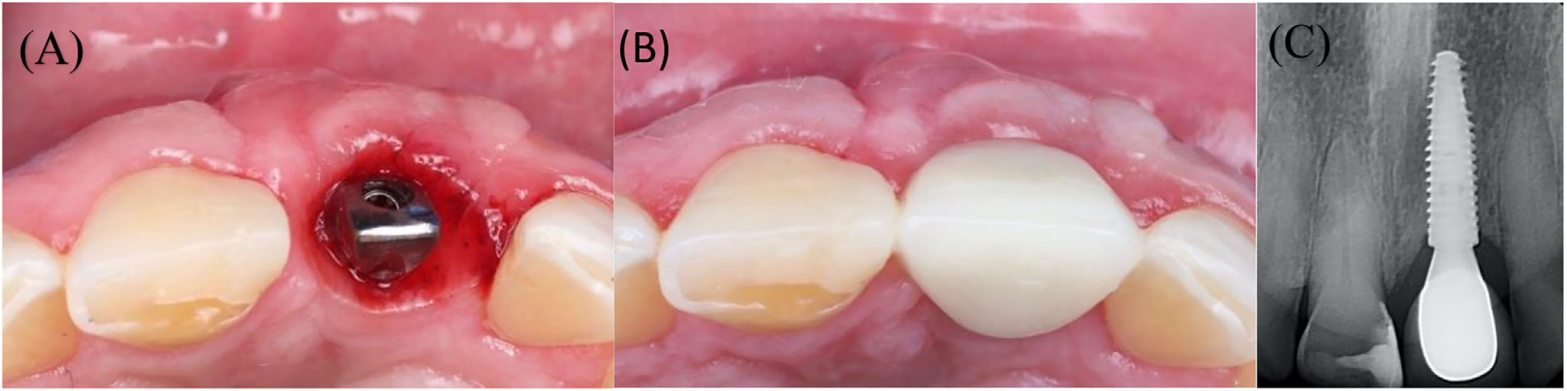
Occlusal view of (A) final abutment, (B) occlusal view of the final crown cemented, and (C) radiograph with the final restoration 8 months after surgery.

**FIGURE 11:**
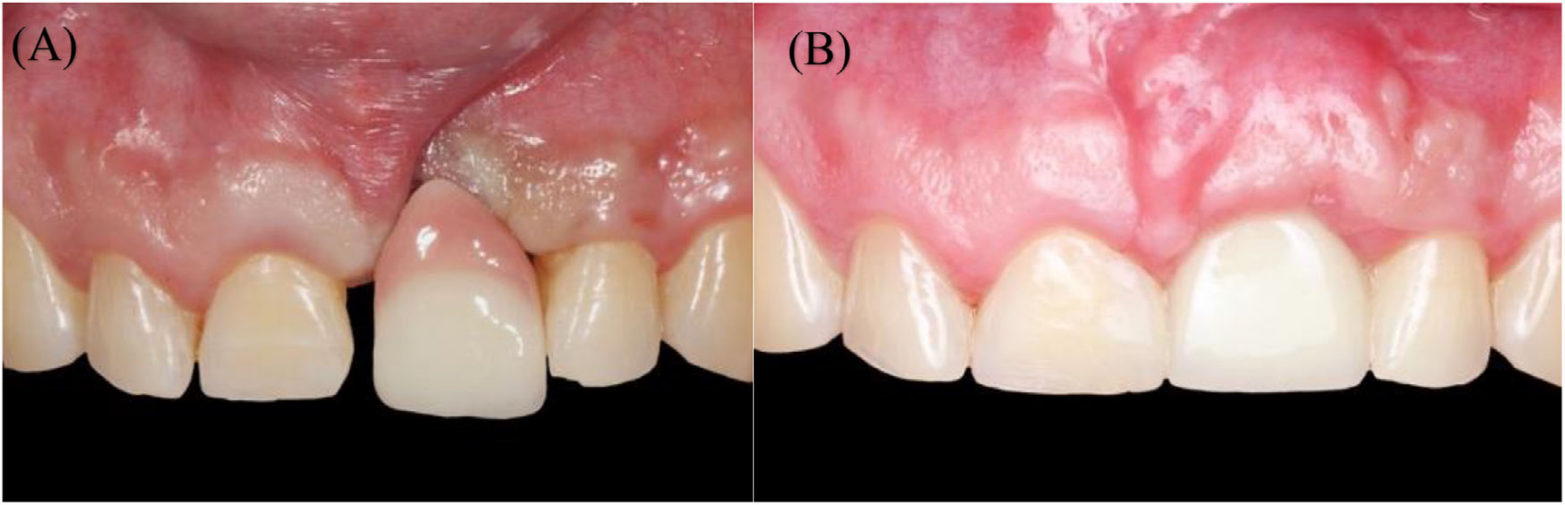
(A) Before treatment. (B) 12 months after final restoration.

After consultation, the patient and his provider agreed on restoring tooth #8 with a direct resin restoration to close the diastema. Eight months after surgery, the implant was restored with a final abutment and cemented crown (Figure 10). The last follow‐up appointment was conducted 12 months post‐restorationand; it was observed that there was no change in the peri‐implant mucosal margin level of #9 (Figure 11).

## DISCUSSION

Predisposing factors contributing to PSTDs include a buccally positioned implant, osseous dehiscence or fenestration of the buccal bone, a thin gingival biotype, minimal or absent keratinized mucosa, vigorous tooth brushing, and an over‐contoured prosthesis.^5^ Among these factors, the buccolingual positioning of the implant is considered the most crucial determinant.^6^, ^7^

Treatment guidelines and decision trees have been proposed in the literature for addressing PSTD,^1^, ^4^, ^8^ with recent emphasis on the quality and dimensions of interproximal tissues and the bucco‐lingual position of the implant as key factors influencing the treatment approach.^4^

Submerged and non‐submerged treatment modalities have been proposed,^4^ depending on the position of the implant platform and the condition of the interproximal soft tissue. In submerged healing, treatment is carried out after the removal of the abutment and crown, allowing the implant and graft to heal without any transmucosal prosthetic components. In non‐submerged healing, the implant abutment and crown are modified and remain in place throughout the grafting and healing process, representing a combined prosthetic‐surgical approach.

In this case, given that the implant had not yet been restored with a final restoration. And when wide, thick interproximal tissues were present, the decision was made to proceed with a combined prosthetic‐surgical approach. A CAF design was selected as it provides greater control over reflecting the buccal tissue in a partial dissection manner, allowing better ability to coronally advance the tissues.

Additionally, a tCTG harvested from the maxillary tuberosity—rich in lamina propria and fibrous connective tissue—has demonstrated superior results in increasing keratinized tissue width and enhancing soft tissue thickness, making it an ideal donor site.^9^ These benefits outweigh the risks of potential esthetic complications reported with tuberosity grafts. Alternatively, a CTG from the palate can be utilized. Other treatment modalities reported in the literature include free gingival grafts,^10^ the VISTA technique,^11^ and tunnel techniques.^12^ A recent study found that a CAF combined with CTG is superior to the tunnel technique in combination with CTG.^13^

A limiting factor in CAF in this case was the height of the distal papilla of #9. As a result, a slight discrepancy in the gingival margin level between the implant and the contralateral tooth persisted after treatment. However, a significant improvement of the peri‐implant mucosal margin level, along with an increase in tissue thickness and keratinization, was achieved. Patient satisfaction was assessed through questions regarding esthetic appearance, gingival color, shape, and symmetry, all of which were reported satisfactory by the patient.

## CONCLUSION

A combined prosthetic‐surgical approach using CAF with tCTG constitutes a valid option for treatment of PSTD class III C in cases where there is an abundant presence of interproximal tissue.

## AUTHOR CONTRIBUTIONS

**Omran B. Zeino**: Conceptualization (lead); writing—original draft (lead). **Yoon J. Kim**: Writing—review & editing.

## CONFLICT OF INTEREST STATEMENT

The authors declare no conflicts of interest.

## Data Availability

Data sharing is not applicable to this article as no datasets were generated or analyzed during the current study.
